# A novel and efficient method to prepare 2-aryltetrahydrofuran-2-ylphosphonic acids

**DOI:** 10.3762/bjoc.6.63

**Published:** 2010-06-09

**Authors:** Vsevolod V Komissarov, Anatoly M Kritzyn, Jouko J Vepsäläinen

**Affiliations:** 1 Engelhardt Institute of Molecular Biology, Russian Academy of Sciences, Vavilov St., 32, Moscow 119991, Russia; 2 Department of Biosciences, Biocenter Kuopio, University of Eastern Finland, P.O. Box 1627, FIN-70211, Kuopio, Finland

**Keywords:** 4-chloro-1-aryl-1-butanones, cyclization, furan, heterocycle, phosphonic acids

## Abstract

A novel one-pot method was developed for the synthesis of the title compounds starting from 4-chloro-1-aryl-1-butanones **1**, phosphorus trichloride and acetic acid. The end products **2** were obtained in 20–94% yield. The cyclization step under acidic conditions probably occurs as a result of anchimeric assistance of the phosphonic acid group.

## Introduction

Our laboratory has conducted systematic evaluation of polymethylene derivatives of nucleic bases with various terminal functional groups [1–4]. As a part of our ongoing project, it seemed important to synthesize compounds with phosphonate and phenyl groups at one end of the hydrocarbon chain and a nucleic base residue at the other end. Previously, it was shown that compounds with similar structures inhibit purine nucleoside phosphorylase and thymidine phosphorylase, enzymes which have been targeted in a number of serious diseases [5–7].

**Figure 1:**
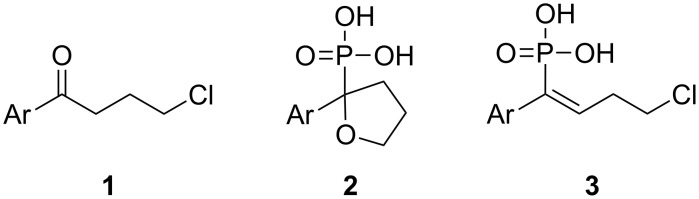
Treatment of 4-chloro-1-aryl-1-butanones **1** with PCl_3_/AcOH lead to unexpected cyclic product **2** instead of the expected alkene **3**.

Recently, we prepared nucleic base derivatives with a terminal Ph-CO-group by alkylation methods starting from 4-chloro-1-aryl-1-butanones **1** [8]. According to the studies of Conant et al. [9–10] conducted about 90 years ago, the target phenylvinylphosphonic acids **3** could be obtained from **1** after treatment with PCl_3_ and AcOH, followed by the elimination of water (Figure 1). After the reduction of the double bond [11], these compounds could then be used for nucleic bases alkylation as described earlier.

## Results and Discussion

**Scheme 1:**
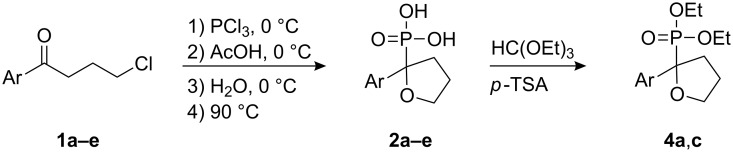
Preparation of cyclic furans **2a–e** and ethyl esters **4a**,**c** from chloroarylbutanones **1**.

In this study, we employed the above noted reaction sequence with our model compound, 4-chloro-1-phenyl-1-butanone (**1a**) [5]. However, the only product formed in good yield was 2-phenyltetrahydrofuran-2-ylphosphonic acid (**2a**) instead of the expected acyclic product. Previously, to obtain phenylvinylphosphonic acids **3**, Conant [9] heated the reaction mixture under atmospheric pressure at high temperature (~280 °C). Initially, we also followed this procedure, but soon realized that the yield of phosphonic acid **2a** was significantly higher under the milder conditions shown in Scheme 1. The yields of the prepared cyclic compounds **2b–e** are summarized in Table 1.

**Table 1:** Prepared compounds **2a–e** with yields.

Aryl group	Product	Yield [%]

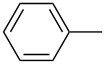	**2a**	94
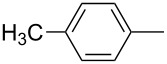	**2b**	59
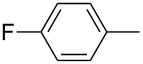	**2c**	83
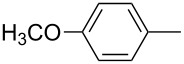	**2d**	57
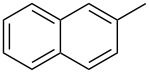	**2e**	20^a^

^a^Isolated via the ammonium salt.

The synthesized phosphonic acids **2** can be easily converted into diethyl esters **4** by refluxing in triethyl orthoformate in the presence of *p*-TSA [12]. Previously, these esters **4** were prepared directly from **1a–d** by refluxing in an excess of P(OEt)_3_. However, this procedure provides high yields of 2-aryltetrahydrofuran-2-ylphosphonic acid esters **4** only if the starting ketones have electron donating substituents at the para-position of the aromatic ring [13–14].

The structures of phosphonic acids **2** were established from MS and NMR spectral data. The cyclic structures of the end products were verified not only from molecular ion peaks in the mass spectra but also from ^1^H NMR data, in which three CH_2_-groups give rise to six chemical shifts. This phenomenon exists if a chiral center (sometimes a prochiral center) is near a CH_2_-group or when a CH_2_ group is a part of a cyclic structure. In this case, the chemical shift difference between the furan ring CH_2_ protons were 0.1–0.5 ppm, clear evidence for the existence of a cyclic structure. The complicated but characteristic ^1^H NMR spectra were analyzed by PERCH software [15]. In addition, ^13^C NMR spectra confirmed the presence of the tetrahydrofuran ring system since characteristic ^1–3^*J*_CP_ coupling constants were found for all four CH_2_ carbons at ~84, ~69, ~36, and ~26 ppm. ^31^P NMR signals of the phosphonic acids **2** were at ca 23 ppm.

**Scheme 2:**
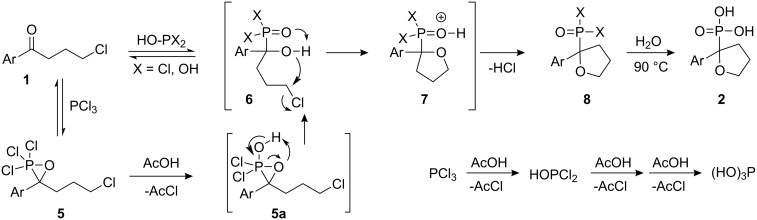
Proposed reaction mechanism for the cyclization reaction.

In order to understand the mechanism of the reaction, we conducted the reactions of **1a** and PCl_3_ in the absence of AcOH and with phosphorous acid in AcOH solution. In the first case, there was no evidence of that any reaction had occurred, whereas in the second case cyclization was observed only under reflux conditions; however, the yield of **2a** was extremely low. Accordingly, in the initial step either PCl_3_ reacts with ketone **1** to form the adduct **5**, as proposed by Conant [9], which is then converted to intermediate **6** via **5a** by the addition of AcOH, or alternatively, HOPCl_2_ (and/or (HO)_2_PCl), formed after acetic acid addition to the mixture, reacts directly with the ketone **1** to produce intermediate **6** as shown in Scheme 2.

The intermediate **6** rapidly forms ring **8** possibly via intermediate **7**. However, the cyclization reaction in which hydroxyl group and chlorine atom are sterically near to each other are normally conducted under basic conditions [16] or at elevated temperatures [17]. A plausible explanation for the cyclization occurring at room temperature without an alkoxide intermediate would be to invoke anchimeric assistance of the P=O group, which could serve as a general base to facilitate cyclization leading to the formation of **7** [18].

**Scheme 3:**
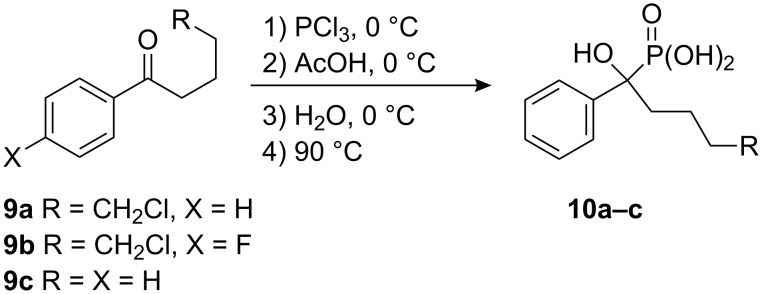
Acyclic products are obtained from pentanones **9a,b** and butanone **9c**.

The length of hydrocarbon chain is also of major importance, since cyclic tetrahydropyran derivatives are not formed in the case of arylpentanones **9a,b** under the same conditions as those used for the synthesis of furans **2** (Scheme 3). According to the NMR spectra, the only phosphorus containing products in the reaction mixtures were the corresponding α-hydroxyphosphonic acids **10**. Similarly, acid **10c** is formed from phenylbutanone **4c** in 41% yield.

In summary, a direct and efficient route was developed for the synthesis of 2-aryltetrahydrofuran-2-ylphosphonic acids – compounds containing a relatively exotic motif – via a one-pot experimental procedure from 4-chloro-1-aryl-1-butanones. The cyclic structure of the synthesized compounds was reliably established by means of various NMR experiments. A mechanism for the cyclization is proposed and it is suggested, that the crucial feature of the reaction is the anchimeric assistance of the P=O group. We have also demonstrated that the tetrahydropyran analogs of the title phosphonic acids were not formed under similar conditions.

## Experimental

**General remarks.** ^1^H, ^13^C and ^31^P NMR spectra were recorded on a Bruker Avance 500 DRX instrument at 500 MHz, 125 MHz and 162 MHz, respectively. Chemical shifts (δ) are reported in ppm relative to tetramethylsilane as internal standard for protons and carbon atoms, and to H_3_PO_4_ (85%) for phosphorus chemical shifts. Exact chemical shifts and coupling constants for protons were calculated using PERCH software [15]. Mass spectra were obtained on an Applied Biosystems/MDS Sciex QSTAR XL spectrometer using ESI technique.

**General procedure for the synthesis of compounds 2a–e:** PCl_3_ (1.2 mL, 13.75 mmol) was added dropwise to 4-chloro-1-aryl-1-butanone (10 mmol) with stirring at 0 °C. Cooling was removed and the reaction mixture stirred at room temperature for 30 min followed by dropwise addition of glacial acetic acid (1.72 mL, 30 mmol) with stirring at 0 °C. Stirring was then continued without cooling for 20 h, ice (50 g) was added and the reaction mixture heated slowly over a water bath to 90 °C. After 40 min at this temperature, the solvents were evaporated in vacuo and the resulting oil was re-evaporated with water (3 × 20 mL). The crystalline residue was washed with cold water (20 mL) and benzene (40 mL). The residue was dried under vacuum (P_2_O_5_ and paraffin) to yield the target acids **2a–d** in 57–94% yield.

**2-Phenyltetrahydrofuran-2-ylphosphonic acid (2a):** ^1^H NMR (DMSO-*d*_6_): δ_H_ 7.5 (2H, bs), 7.450 (2H, d, ^3^*J*_HH_ = 7.80 Hz), 7.288 (2H, dd, ^3^*J*_HH_ = 7.80, 7.39 Hz), 7.203 (1H, dd), 3.923 (1H, ddd, ^2^*J*_HH_ = −7.84 Hz, ^3^*J*_HH_ = 7.11, 6.90), 3.847 (1H, ddd, ^2^*J*_HH_ = −7.84 Hz, ^3^*J*_HH_ = 7.37, 5.42 Hz), 2.599 (1H, dddd, ^3^*J*_HP_ = 15.58 Hz, ^2^*J*_HH_ = −12.54 Hz, ^3^*J*_HH_ = 8.13, 7.82 Hz), 2.137 (1H, dddd, ^3^*J*_HP_ = 10.97 Hz, ^2^*J*_HH_ = −12.54 Hz, ^3^*J*_HH_ = 7.77, 5.12 Hz), 1.980 (ddddd, ^2^*J*_HH_ = −11.74 Hz, ^3^*J*_HH_ = 8.13, 6.90, 5.42, 5.12), 1.660 (ddddd, ^3^*J*_HH_ = 7.82, 7.77, 7.37, 7.11); ^13^C NMR (DMSO-*d*_6_): δ_C_ 142.4 s, 127.3 s, 126.4 s, 126.3 s, 83.6 (d, *J*_CP_ = 167.0 Hz), 68.4 (d, *J*_CP_ = 6.4 Hz), 35.7 s, 25.5 (d, *J*_CP_ = 4.8 Hz); ^31^P NMR (DMSO-*d*_6_): δ_P_ 23.11; MS (ESI): *m/z* 227 [M^+^−H].

**2-(4-Methylphenyl)tetrahydrofuran-2-ylphosphonic acid (2b):** ^1^H NMR (DMSO-*d*_6_): δ_H_ 8.6 (2H, bs), 7.326 (2H, d, ^3^*J*_HH_ = 7.87 Hz), 7.089 (2H, d), 3.906 (1H, ddd, ^2^*J*_HH_ = −7.81 Hz, ^3^*J*_HH_ = 7.13, 6.94), 3.831 (1H, ddd, ^2^*J*_HH_ = −7.81 Hz, ^3^*J*_HH_ = 7.40, 5.36 Hz), 2.270 (3H, s), 2.567 (1H, dddd, ^3^*J*_HP_ = 15.51 Hz, ^2^*J*_HH_ = −12.51 Hz, ^3^*J*_HH_ = 8.09, 7.96 Hz), 2.110 (1H, dddd, ^3^*J*_HP_ = 10.78 Hz, ^2^*J*_HH_ = −12.51 Hz, ^3^*J*_HH_ = 7.71, 4.98 Hz), 1.964 (ddddd, ^2^*J*_HH_ = −11.73 Hz, ^3^*J*_HH_ = 8.09, 6.94, 5.36, 4.98), 1.648 (ddddd, ^3^*J*_HH_ = 7.96, 7.70, 7.40, 7.13); ^13^C NMR (DMSO-*d*_6_): δ_C_ 139.7 (d, *J*_CP_ = 5.6 Hz), 135.9 (d, *J*_CP_ = 2.4 Hz), 128.4 s, 126.6 (d, *J*_CP_ = 3.2 Hz), 83.9 (d, *J*_CP_ = 169.0 Hz), 68.8 (d, *J*_CP_ = 6.4 Hz), 36.0 (d, *J*_CP_ = 2.4 Hz), 25.9 (d, *J*_CP_ = 4.8 Hz), 20.9 s; ^31^P NMR (DMSO-*d*_6_): δ_P_ 23.56; MS (ESI): *m/z* 241 [M^+^−H].

**2-(4-Fluorophenyl)tetrahydrofuran-2-ylphosphonic acid (2c):** ^1^H NMR (DMSO-*d*_6_): δ_H_ 8.3 (2H, bs), 7.463 (2H, dd, ^3^*J*_HH_ = 8.71 Hz, ^4^*J*_HF_ = 5.58 Hz), 7.111 (2H, dd, ^3^*J*_HF_ = 8.01 Hz), 3.919 (1H, ddd, ^2^*J*_HH_ = −7.87 Hz, ^3^*J*_HH_ = 7.16, 6.90 Hz), 3.854 (1H, ddd, ^2^*J*_HH_ = −7.87 Hz, ^3^*J*_HH_ = 7.39, 5.36 Hz), 2.588 (1H, dddd, ^3^*J*_HP_ = 15.41 Hz, ^2^*J*_HH_ = −12.58 Hz, ^3^*J*_HH_ = 8.11, 7.90 Hz), 2.126 (1H, dddd, ^3^*J*_HP_ = 10.86 Hz, ^2^*J*_HH_ = −12.58 Hz, ^3^*J*_HH_ = 7.75, 5.07 Hz), 1.986 (ddddd, ^2^*J*_HH_ = −11.77 Hz, ^3^*J*_HH_ = 8.11, 6.90, 5.36, 5.07), 1.667 (ddddd, ^3^*J*_HH_ = 7.90, 7.75, 7.39, 7.16); ^13^C NMR (DMSO-*d*_6_): δ_C_ 161.9 (d, *J*_CF_ = 242.6 Hz), 138.9 s, 128.9 (d, *J* = 4.6 Hz), 114.6 (d, *J* = 21.4 Hz), 83.9 (d, *J*_CP_ = 169.4 Hz), 69.2 (d, *J*_CP_ = 6.1 Hz), 36.2 s, 26.1 (d, *J*_CP_ = 4.6 Hz); ^31^P NMR (DMSO-*d*_6_): δ_P_ 22.72; MS (ESI): *m/z* 245 [M^+^−H].

**2-(4-Methoxyphenyl)tetrahydrofuran-2-ylphosphonic acid (2d):** ^1^H NMR (DMSO-*d*_6_): δ_H_ 8.3 (2H, bs), 7.357 (2H, d, ^3^*J*_HH_ = 8.55 Hz), 6.857 (2H, d), 3.909 (1H, dt, ^2^*J*_HH_ = −7.77 Hz, ^3^*J*_HH_ = 7.06), 3.837 (1H, ddd, ^2^*J*_HH_ = −7.77 Hz, ^3^*J*_HH_ = 7.35, 5.33 Hz), 3.730 (3H, s), 2.562 (1H, dddd, ^3^*J*_HP_ = 15.32 Hz, ^2^*J*_HH_ = −12.48 Hz, ^3^*J*_HH_ = 8.06, 8.04 Hz), 2.118 (1H, dddd, ^3^*J*_HP_ = 10.69 Hz, ^2^*J*_HH_ = −12.48 Hz, ^3^*J*_HH_ = 7.64, 4.98 Hz), 1.974 (ddddd, ^2^*J*_HH_ = −11.70 Hz, ^3^*J*_HH_ = 8.04, 7.06, 5.33, 4.98), 1.666 (ddddd, ^3^*J*_HH_ = 8.06, 7.64, 7.35, 7.06); ^13^C NMR (DMSO-*d*_6_): δ_C_ 158.6 s, 134.5 (d, *J*_CP_ = 4.8 Hz), 127.9 s, 113.3 s, 83.7 (d, *J*_CP_ = 169.5 Hz), 68.8 (d, *J*_CP_ = 6.4 Hz), 55.4 s, 35.9 s, 25.9 (d, *J*_CP_ = 4.8 Hz); ^31^P NMR (DMSO-*d*_6_): δ_P_ 23.74; MS (ESI): *m/z* 257 [M^+^−H].

**2-(Naphthalen-2-yl)tetrahydrofuran-2-ylphosphonic acid (2e):** After the solvents were evaporated, an excess of semi-saturated methanolic ammonia was added and the mixture evaporated in vacuo. The crystalline product was washed with benzene (70 mL), the residue transferred to a separation funnel and HCl (15%, 50 mL) and CH_2_Cl_2_ (50 mL) added. The organic layer was separated and the aqueous phase extracted with CH_2_Cl_2_ (4 × 50 mL). The combined organic extracts were dried (Na_2_SO_4_), evaporated in vacuo and the product recrystallized from benzene – heptane (2:1 mixture, 30 mL) to give **2e**: ^1^H NMR (DMSO-*d*_6_): δ_H_ 9.23 (2H, bs), 8.01 (1H, s), 7.90 (1H, d, *J* = 7.2 Hz), 7.85 (2H, m), 7.70 (1H, d, *J* = 8.7 Hz), 7.46 (2H, m), 4.01 (1H, m), 3.92 (1H, m), 2.73 (1H, m), 2.26 (1H, m), 2.00 (1H, m), 1.67 (1H, m); ^13^C NMR (DMSO-*d*_6_): δ_C_ 140.4 (d, *J*_CP_ = 5.6 Hz), 132.7 (d, *J*_CP_ = 2.4 Hz), 132.3 (d, *J*_CP_ = 1.6 Hz), 128.1 s, 127.5 s, 127.0 s, 126.1 s, 125.8 s, 125.5 (d, *J*_CP_ = 2.4 Hz), 124.9 (d, *J*_CP_ = 5.6 Hz), 84.1 (d, *J*_CP_ = 167.0 Hz), 68.8 (d, *J*_CP_ = 6.4 Hz), 35.8 (d, *J*_CP_ = 3.2 Hz), 25.8 (d, *J*_CP_ = 4.8 Hz); ^31^P NMR (DMSO-*d*_6_): δ_P_ 22.98; MS (ESI): *m/z* 277 [M^+^−H].
